# Infrared and Raman spectra of Bi_2_O_2_X and Bi_2_OX_2_ (X = S, Se, and Te) studied from first principles calculations†

**DOI:** 10.1039/c9ra02584g

**Published:** 2019-06-10

**Authors:** Yao-Di Xu, Cong Wang, Yang-Yang Lv, Y. B. Chen, Shu-Hua Yao, Jian Zhou

**Affiliations:** National Laboratory of Solid State Microstructures and Department of Materials Science and Engineering, Nanjing University Nanjing 210093 China zhoujian@nju.edu.cn; National Laboratory of Solid State Microstructures and Department of Physics, Nanjing University Nanjing 210093 China; Collaborative Innovation Center of Advanced Microstructures, Nanjing University Nanjing 210093 China; Jiangsu Key Laboratory of Artificial Functional Materials, Nanjing University Nanjing 210093 China

## Abstract

The bismuth oxychalcogenide compounds contain many different kinds of materials, such as Bi_2_O_2_X and Bi_2_OX_2_ (X = S, Se, and Te). These materials have different but similar layered crystal structures and exhibit various interesting physical properties. Here, we have theoretically investigated their Raman and infrared spectra by first principles calculations based on density functional theory. It is found that in Bi_2_O_2_Se the calculated frequency of the A_1g_ Raman active mode is in good agreement with the experimental measurements while the other three modes are ambiguous or not observed yet. The Raman and infrared spectra of other materials are also presented and need further confirmation. Our work provides the structural fingerprints of these materials, which could be helpful in identifying the crystal structures in future experiments.

## Abstract 2

Crystal structures of bismuth oxychalcogenide compounds Bi_2_O_2_X and Bi_2_OX_2_ (X = S, Se, and Te).

## Graphical abstract

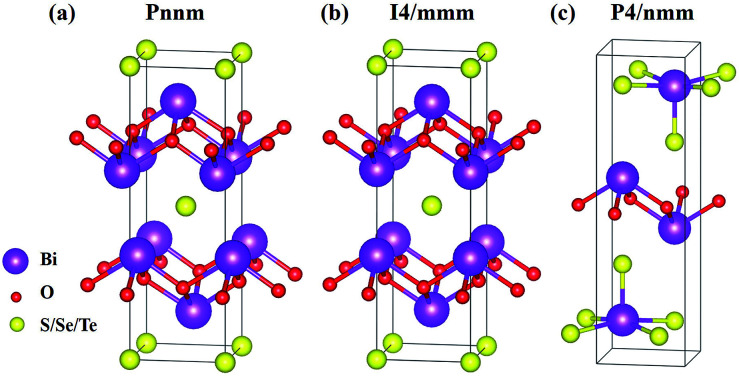

## 1 Introduction

In recent years, bismuth oxychalcogenide materials Bi–O–X (X = S, Se, and Te) have attracted more and more attention. Among these materials, Bi_2_O_2_Se, synthesized more than forty years ago,^1^ is one of the most studied materials and has now become a very hot topic due to its various and interesting physical properties. First, Bi_2_O_2_Se was suggested to be a good thermoelectric material.^2–7^ In 2010, Ruleova *et al.* reported the thermoelectric properties of Bi_2_O_2_Se and they found that Bi_2_O_2_Se is an n-type semiconductor with a very low thermal conductivity and a relatively high figure of merit *ZT* about 0.2 at 800 K.^2^ Several theoretical works were also conducted to explore its thermoelectric properties.^8–11^ Second, Bi_2_O_2_Se has an ultrahigh electron mobility.^12–17^ An earlier work in 2012 found that the room temperature Hall mobility of Bi_2_O_2_Se single crystal was on the order of 300 cm^2^ s^−1^ V^−1^.^12^ Recently, it was found that the low temperature (about 2 K) Hall mobility can reach more than 2.0 × 10^4^ cm^2^ s^−1^ V^−1^ in Bi_2_O_2_Se thin film^13–15^ and 4.0 × 10^4^ cm^2^ s^−1^ V^−1^ in Bi_2_O_2_Se single crystal.^16^ Very recently, we have observed a superior Hall mobility of 2.2 × 10^5^ cm^2^ s^−1^ V^−1^ at 2 K in a high quality Bi_2_O_2_Se single crystal.^17^ The high mobility in Bi_2_O_2_Se is possibly due to the self-modulation doping, *i.e.* the electron donor states lie above the lowest conduction band, not in the middle of the band gap.^18^ Furthermore, high mobility usually induces a large magnetoresistance (MR),^19^ which was also observed in Bi_2_O_2_Se. A longitudinal MR of about 600% (at 15 Tesla and 2 K) and 9000% (at 9 Tesla and 2 K) in Bi_2_O_2_Se single crystals was observed in two recent experiments.^16,17^ Third, due to its high mobility and suitable band gap (about 0.8 eV), Bi_2_O_2_Se was used in optoelectronic devices and infrared (IR) photo-detectors.^20–22^

Bi_2_O_2_Te has the same crystal structure as that of Bi_2_O_2_Se, but it is much less studied. Luu and Vaqueiro found that Bi_2_O_2_Te ceramics is an n-type semiconductor with a smaller band gap (0.23 eV), electron mobility (47 cm^2^ s^−1^ V^−1^ at room temperatures), and *ZT* (0.13 at 573 K), compared with those of Bi_2_O_2_Se.^23^ The similar compound Bi_2_O_2_S is also less studied. Bi_2_O_2_S was first synthesized in 1984 and it has a different crystal structure to that of Bi_2_O_2_Se.^24^ There are only a few studies on its optical properties.^25–27^ For example, it was found that Bi_2_O_2_S has an indirect band gap of 1.12 eV and it is an excellent photoelectric material.^27^

On the other hand, there is another kind of bismuth oxychalcogenides Bi_2_OX_2_ (X = S, Se, and Te), which all share the same tetragonal lattice system. Among them, Bi_2_OS_2_ has been experimentally synthesized recently and it was a candidate as an optoelectronic material in the near-IR region.^28^ First principles calculations indicated that the two-dimensional Bi_2_OS_2_ nanosheet possesses a direct band gap and an ultrahigh electron mobility (up to 2.6 × 10^4^ cm^2^ s^−1^ V^−1^).^29^ To the best of our knowledge, Bi_2_OSe_2_ and Bi_2_OTe_2_ have not been synthesized experimentally. However, first principles calculations showed that they have the same crystal structure as that of Bi_2_OS_2_.^30^ In particular, the calculated electron and hole effective mass of Bi_2_OX_2_ is very small. For example, the effective mass of Bi_2_OTe_2_ is only 0.02 and 0.012 for electron and hole.^30^ Another theoretical study indicated that Bi_2_OX_2_ materials show promising characteristics in applications for solar cells and thermoelectric devices.^31^

Besides Bi_2_O_2_X and Bi_2_OX_2_, the first BiS_2_ family superconductor Bi_4_O_4_S_3_ was studied over the past few years.^32,33^ Later, it was found that Bi_4_O_4_S_3_ is a mixture of the two phases, Bi_2_OS_2_ and Bi_3_O_2_S_3_.^34^ The former is non-superconducting, while the latter is superconducting.^34–36^

Therefore, we can see that the Bi–O–X system contains many kinds of materials with various interesting physical properties. From the experimental point of view, it is of course very important to identify the structure of the grown crystal from the many similar Bi–O–X materials. In this regard, Raman and IR spectra are convenient and powerful methods to provide the structural fingerprints of materials. However, we find that the Raman and IR studies of these materials are quite lacking. Only a few works about the Raman spectra of Bi_2_O_2_Se and Bi_2_O_2_Te have been reported until now.^14,16,37,38^ For this reason, we have systematically calculated the phonon, irreducible representations, Raman and IR spectra, vibrational eigenvectors of optical phonons, and polarized Raman configurations of six materials: Bi_2_O_2_X and Bi_2_OX_2_. We mainly present the results of Bi_2_O_2_Se and Bi_2_O_2_Te since they can be compared with other works. The Raman and IR spectra of the other four materials are also given briefly and could be referenced by future experiments.

## 2 Computational details

The vibrational properties of Bi_2_O_2_X and Bi_2_OX_2_ (X = S, Se, and Te) are calculated by density functional theory (DFT) implemented in the Vienna *ab initio* simulation package (VASP).^39,40^ The projected augmented wave method^41,42^ and the generalized gradient approximation with the Perdew–Burke–Ernzerhof exchange–correlation functional^43^ are used. The DFT-D3 method^44,45^ is used to correct the van der Waals interactions in these layered materials. The plane-wave cutoff energy is 520 eV for all materials. Both the internal atomic positions and the lattice constants are allowed to relax until the maximal residual Hellmann–Feynman forces on atoms are smaller than 0.002 eV Å^−1^. The *k*-mesh is 8 × 8 × 2 for Bi_2_O_2_S and Bi_2_OX_2_ and 8 × 8 × 8 for Bi_2_O_2_Se and Bi_2_O_2_Te. The Phonopy package^46^ is used to calculate the phonon frequencies, eigenvectors and irreducible representations of the materials. The crystal structures and eigenvectors are plotted by the VESTA program.^47^

The IR and Raman activity of phonon modes can be analyzed by their irreducible representations. However their intensities need additional calculations. The IR intensity of a phonon mode is given by the corresponding oscillator strength:^48^
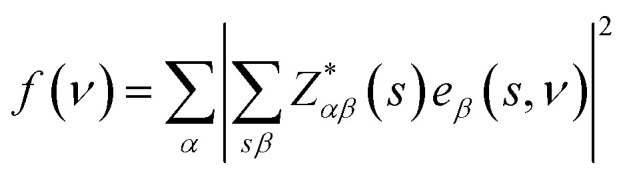
where the *e*_*β*_(*s*,*v*) is the normalized vibrational eigenvector of the *ν*th phonon mode of the *s*th atom in the unit cell. *α* and *β* are the Cartesian coordinates: *x*,*y*,*z*. 
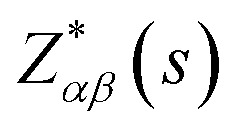
 is the Born effective charge tensor of the *s*th atom. The Born effective charge tensor and the phonon eigenvectors are calculated by the density functional perturbation theory (DFPT) implemented in the VASP code. This method has been applied to different material systems.^48–51^

The off-resonance Raman intensity of a phonon mode can be estimated by calculating the derivative of the macroscopic dielectric tensor with respect to the normal mode coordinate:^52^
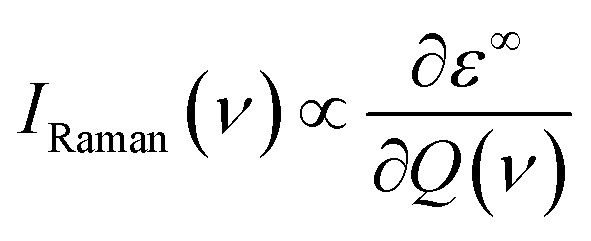
where the *ε*^∞^ is the macroscopic high-frequency dielectric constant and *Q*(*v*) is the normal mode coordinate of the *ν*th phonon mode. In practice, the derivative is replaced by the central difference based on the macroscopic dielectric matrix evaluated at positive and negative displacement along the phonon mode *Q*(*v*). The macroscopic dielectric matrix is also calculated by the DFPT method in the VASP code. This method has also been applied to different material systems.^53,54^

## 3 Results and discussions

### 3.1 Crystal structures of Bi_2_O_2_X and Bi_2_OX_2_

**Fig. 1:**
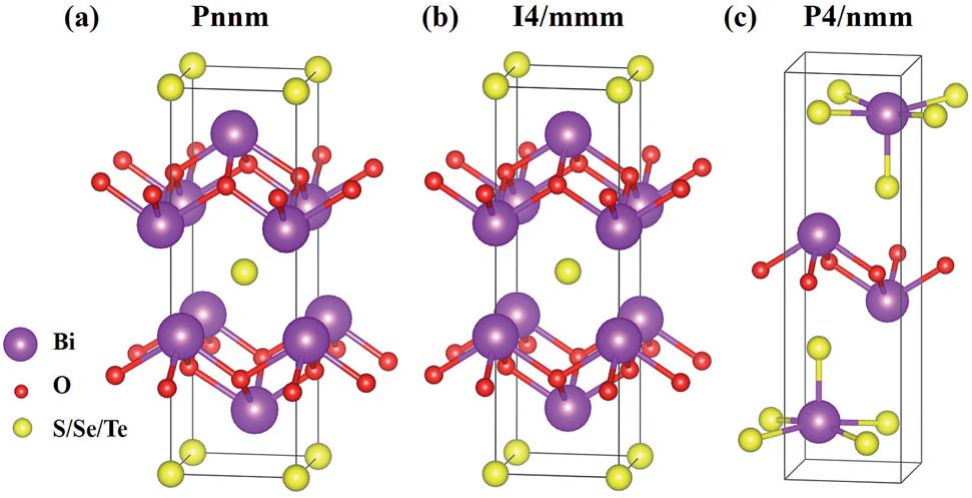
Layered crystal structures of (a) orthorhombic Bi_2_O_2_S, (b) tetragonal Bi_2_O_2_Se and Bi_2_O_2_Te, (c) tetragonal Bi_2_OS_2_, Bi_2_OSe_2_, and Bi_2_OTe_2_. The purple, red, and yellow balls represent Bi, O, and S/Se/Te atoms respectively.

The six materials Bi_2_O_2_X and Bi_2_OX_2_ (X = S, Se, and Te) have three different crystal structures. Bi_2_O_2_S belongs to a primitive orthorhombic lattice with a space group *Pnnm* (no. 58),^24^ while Bi_2_O_2_Se and Bi_2_O_2_Te possess a body centered tetragonal lattice with a space group *I*4/*mmm* (no. 139).^1,13,23^ On the other hand, Bi_2_OX_2_ have a primitive tetragonal lattice with a space group *P*4/*nmm* (no. 129).^28,55^ All the materials show layered structures as shown in Fig. 1. Bi_2_O_2_X consists of two Bi_2_O_2_ and two X layers, while Bi_2_OX_2_ is composed of one Bi_2_O_2_ and two BiX_2_ layers in a unit cell. Although the symmetries of Bi_2_O_2_S and Bi_2_O_2_Se are totally different, the structure of Bi_2_O_2_S is a slightly distorted form of Bi_2_O_2_Se.^24^ Therefore, the difference between the two structures shown in Fig. 1(a) and (b) is hardly visible to the naked eye. All the structures shown in Fig. 1 contain ten atoms in the unit cell. However, Bi_2_O_2_Se and Bi_2_O_2_Te shown in Fig. 1(b) is a conventional cell, which in fact contains two primitive cells.

It is noted that among the six materials, to the best of our knowledge, Bi_2_OSe_2_ and Bi_2_OTe_2_ have not been synthesized experimentally. Their crystal structures are predicted to be the same as that of Bi_2_OS_2_ by first principles calculations.^30^

The calculated lattice constants in this work with the DFT-D3 correction are listed in Table 1. It is obvious that our calculated results are well consistent with the experimental measurements with the largest difference less than 1%. Our results are also in good agreement with other theoretical work.^30^

**Table 1:** Calculated lattice constants of Bi_2_O_2_X and Bi_2_OX_2_ (X = S, Se, and Te) in the unit of Å. Other theoretical and experimental results are also given for comparison

Symmetry	Material	Reference	*a*	*b*	*c*
Orthorhombic *Pnnm*	Bi_2_O_2_S	This work	3.837	3.848	11.94
		Experiment^24^	3.840	3.874	11.92
		Theory^30^	3.87	3.89	11.99
Tetragonal *I*4/*mmm*	Bi_2_O_2_Se	This work	3.891	3.891	12.20
		Experiment^1^	3.891	3.891	12.21
		Experiment^13^	3.88	3.88	12.16
		Theory^30^	3.91	3.91	12.38
	Bi_2_O_2_Te	This work	3.984	3.984	12.65
		Experiment^23^	3.980	3.980	12.70
		Theory^30^	4.01	4.01	12.63
Tetragonal *P*4/*nmm*	Bi_2_OS_2_	This work	3.950	3.950	13.84
		Experiment^28^	3.961	3.961	13.80
		Experiment^55^	3.964	3.964	13.83
		Theory^30^	3.96	3.96	13.69
	Bi_2_OSe_2_	This work	4.044	4.044	14.56
		Theory^30^	4.05	4.05	14.46
	Bi_2_OTe_2_	This work	4.193	4.193	15.81
		Theory^30^	4.17	4.17	15.99

With the optimized structures, the zone-centered phonon modes, irreducible representations, IR and Raman spectra of the six materials are calculated. In the following subsections, we first present the detailed results of Bi_2_O_2_Se and Bi_2_O_2_Te since both materials have the same crystal structure and the Raman spectrum of Bi_2_O_2_Se is better studied than other materials. Then the brief results of Bi_2_O_2_S and Bi_2_OX_2_ are also given.

### 3.2 *I*4/*mmm* tetragonal Bi_2_O_2_Se and Bi_2_O_2_Te

The calculated zone-centered optical phonon frequencies of Bi_2_O_2_Se and Bi_2_O_2_Te are listed in Table 2. The highest phonon frequency of Bi_2_O_2_Se is about 433.3 cm^−1^, while it is only 396.1 cm^−1^ in Bi_2_O_2_Te due to the heavier atom mass. Bi_2_O_2_Se and Bi_2_O_2_Te have the same space group of *I*4/*mmm* (point group *D*_4h_), and their irreducible representations at the *Γ* point in the Brillouin zone are:*Γ*_acoustic_ = E_u_ + A_2u_*Γ*_optic_ = 2E_u_ + 2A_2u_ + 2E_g_ + A_1g_ + B_1g_There are five atoms in the primitive cell of Bi_2_O_2_Se, therefore we can find three acoustic and twelve optical modes. These irreducible representations are also assigned to each optical phonon mode as shown in Table 2. According to the character table of the *D*_4h_ point group, the E_u_ and A_2u_ modes are IR active, while the E_g_, A_1g_, and B_1g_ modes are Raman active in Bi_2_O_2_Se and Bi_2_O_2_Te. Therefore, both materials have four Raman active (two double degenerated E_g_ mode and two non-degenerated A_1g_ and B_1g_ modes) and four IR active modes (two double degenerated E_u_ modes and two non-degenerated A_2u_ modes), as indicated in Table 2.

**Table 2:** Calculated frequencies and Mulliken symbols of zone-centered optical phonon modes of Bi_2_O_2_Se and Bi_2_O_2_Te. The theoretical frequencies in other works by Pereira^37^ and Cheng^38^ are also listed for comparison. Raman or IR activity of each mode is also indicated by “Raman” and “IR”. The unit of the phonon frequency is cm^−1^

Symmetry	Bi_2_O_2_Se			Bi_2_O_2_Te		Activity
	This work	Pereira^37^	Cheng^38^	This work	Cheng^38^	
E_u_	54.8	59.2		56.4		IR
A_2u_	65.0	64.5		63.3		IR
E_g_	67.3	72.0	67.99	69.1	67.01	Raman
A_1g_	162.9	165.7	159.89	150.4	147.48	Raman
E_u_	268.0	293.9		243.6		IR
B_1g_	354.3	369.4	364.02	336.0	340.33	Raman
A_2u_	377.8	402.8		347.3		IR
E_g_	433.3	444.0	428.68	396.1	386.15	Raman

Recently, there have been two joint experimental and theoretical works by Pereira *et al.*^37^ and Cheng *et al.*,^38^ in which the phonon frequencies of Bi_2_O_2_Se and Bi_2_O_2_Te are also calculated. We listed their data in Table 2 for comparison. It is found that most of the calculated frequencies are in good agreement with ours, except for the two high-frequency IR active modes (E_u_ and A_2u_) in Bi_2_O_2_Se, which have a maximal discrepancy of about 25 cm^−1^. Phonon frequencies depend on the second derivative of the total energy, therefore the accuracy of the phonon calculation is usually not as good as the ones of the total energy calculations. Many parameters, such as the exchange–correlation functional, will affect the theoretical phonon frequencies. Therefore, we think such differences between these works are acceptable in phonon calculations.

**Fig. 2:**
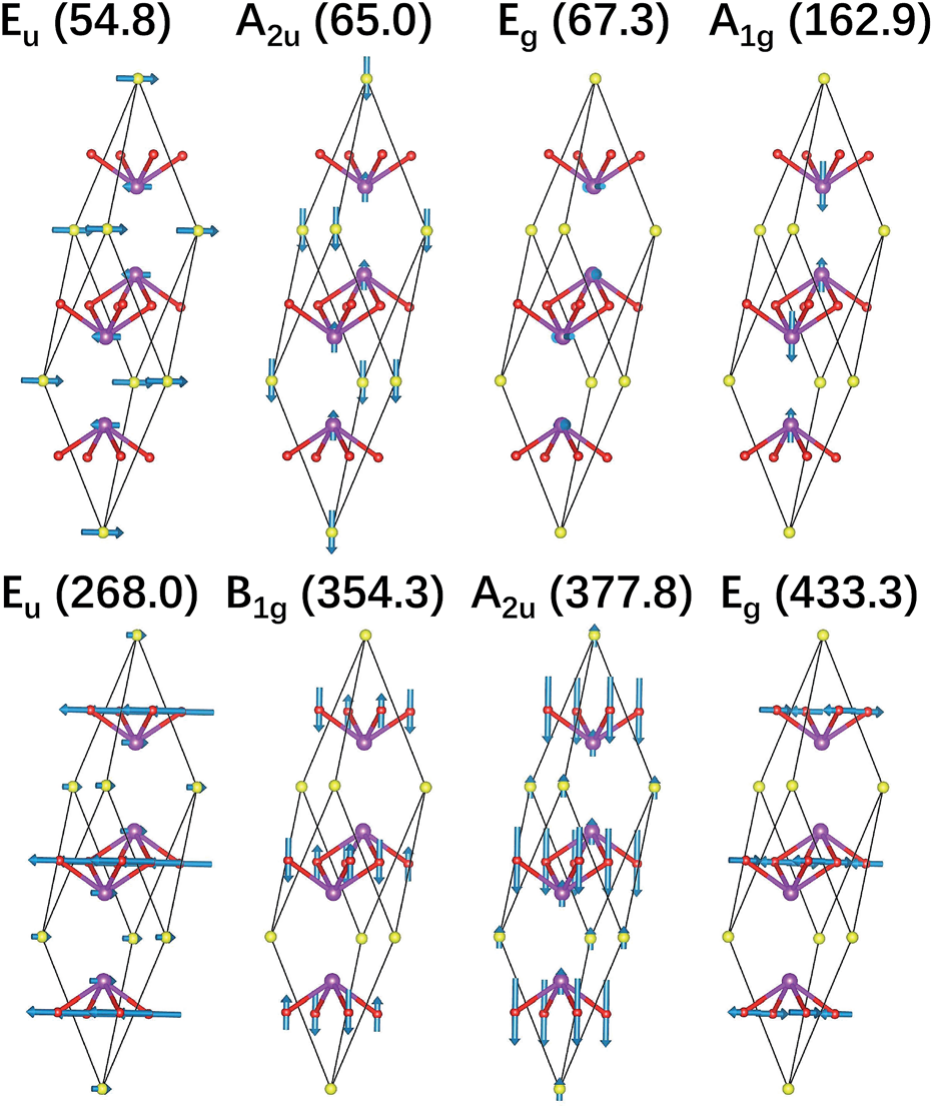
Vibrational eigenvectors of the zone-centered optical phonon modes shown in the primitive cell of Bi_2_O_2_Se. The purple, red, and yellow balls represent Bi, O, and Se atoms respectively.

We also illustrate the vibrational eigenvectors of Bi_2_O_2_Se in Fig. 2. It is found that the two low-frequency Raman active modes (E_g_ and A_1g_) are related to the in-plane and out-of-plane vibrations of Bi atoms, respectively. While the two high-frequency Raman active modes (B_1g_ and E_g_) represent the out-of-plane and in-plane vibrations of O atoms, respectively. Vibrations of Se atoms are not involved in any Raman active modes. The two low-frequency IR active modes (E_u_ and A_2u_) are related to the in-plane and out-of-plane vibrations of Bi and Se atoms, respectively. While the two high-frequency IR active modes (E_u_ and A_2u_) mainly represent the in-plane and out-of-plane vibrations of O atoms, respectively. The vibrational eigenvectors of Bi_2_O_2_Te are similar to those of Bi_2_O_2_Se, which are not shown here.

Then we present a detailed analysis about the polarized configurations for the Raman active modes of Bi_2_O_2_Se and Bi_2_O_2_Te. The Raman tensors of the *D*_4h_ point group can be written as:
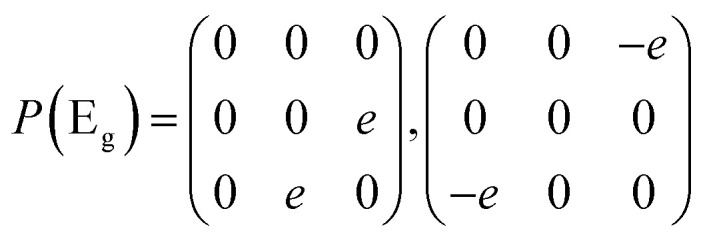

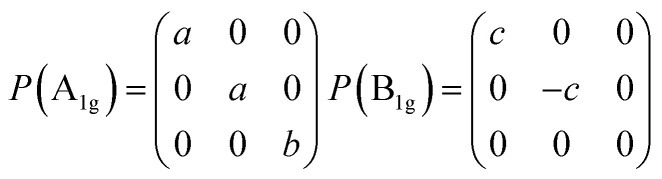
Qualitatively, the Raman intensity *I* of a phonon mode can be calculated by the formula *I* ∞ |*e*_i_·*P*·*e*_s_|^2^, where *e*_i_ and *e*_s_ are polarization directions of the incident and scattered light and *P* is the Raman tensor given above. In Table 3, we present the non-equivalent polarized configurations for the Raman active modes of Bi_2_O_2_Se and Bi_2_O_2_Te. In the configuration notation *A*(*BC*)*D*, *A* and *D* represent the propagation directions of the incident and scattered light respectively, while *B* and *C* represent the polarization directions of the incident and scattered light respectively. In the right angle scattering geometry, the propagation directions of the incident and scattered light are orthogonal (first five configurations in Table 3). In the back scattering geometry, the propagation directions of the incident and scattered light are anti-parallel (last four configurations in Table 3).

**Table 3:** The right angle and back scattering geometries in the polarized configurations of Raman active modes of Bi_2_O_2_Se and Bi_2_O_2_Te. The modes that can be observed in the configuration are indicated by the mark ✓

Configurations	A_1g_	B_1g_	E_g_
*X*(*YY*)*Z*	✓	✓	
*Z*(*XX*)*Y*	✓	✓	
*X*(*ZZ*)*Y*	✓		
*X*(*YZ*)*Y*			✓
*Z*(*XZ*)*X*			✓
−*Z*(*XX*)*Z*	✓	✓	
−*Y*(*XX*)*Y*	✓	✓	
−*X*(*ZZ*)*X*	✓		
−*X*(*YZ*)*X*			✓

From Table 3, it is interesting to find that the E_g_ mode cannot be observed with the A_1g_ and B_1g_ ones simultaneously under the same polarized configuration. Also, only one A_1g_ mode can be observed in the polarized configurations: *X*(*ZZ*)*Y* or −*X*(*ZZ*)*X*. Therefore, all of the Raman active modes can be well identified under different polarized configurations. Of course, in this case, the frequencies of the four Raman active modes in Bi_2_O_2_Se and Bi_2_O_2_Te are well separated and therefore it is quite easy to identify these modes in experiments according to their frequencies without considering their polarized configurations.

**Fig. 3:**
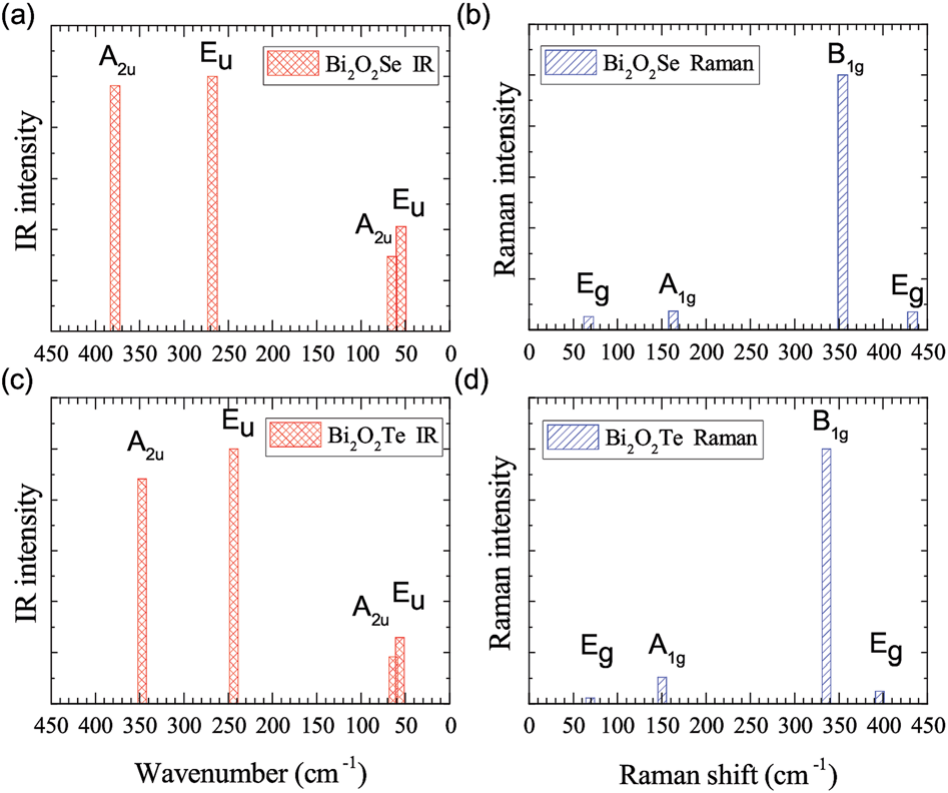
Calculated IR and Raman intensities (arbitrary unit) of Bi_2_O_2_Se and Bi_2_O_2_Te.

IR and Raman intensities of Bi_2_O_2_Se and Bi_2_O_2_Te are also calculated directly by first principles calculations based on the equations in Section II, which are shown in Fig. 3. From Fig. 3(a) and (c), we can see that the two high-frequency IR active modes (E_u_ and A_2u_) have relatively higher intensities than those of the low-frequency modes (E_u_ and A_2u_). On the other hand, in Fig. 3(b) and (d), the Raman active mode B_1g_ has the highest intensity for both materials, while the other three modes have much lower intensities.

Recently, there have been four experimental works,^14,16,37,38^ in which the Raman spectrum of Bi_2_O_2_Se was given. Wu *et al.* have synthesized the atomically thin two-dimensional and the bulk Bi_2_O_2_Se crystals and they observed two Raman peaks located at about 100 and 159 cm^−1^.^14^ Tong *et al.* have grown high-quality Bi_2_O_2_Se single crystals and found two main Raman peaks located at around 90 and 159 cm^−1^, which are associated with the symmetries of E^2^_g_ and A^2^_1g_ respectively.^16^ However, it seems that the E^2^_g_ mode in their Figure is made up of two adjacent peaks located at 84 and 90 cm^−1^.^16^ Pereira *et al.* studied the physical properties of Bi_2_O_2_Se at high pressure, in which they only observed one most intense Raman peak at around 159.2 cm^−1^ at room pressure.^37^ The theoretical low-frequency E_g_ mode (near 70 cm^−1^) can only be observed at high pressure.^37^ Cheng *et al.* have measured the Raman spectra of Bi_2_O_2_Se and Bi_2_O_2_Te.^38^ These results are summarized in Table 4, from which we can see that the Raman active mode A_1g_ at about 160 cm^−1^ can be well confirmed, while the E_g_ mode below 100 cm^−1^ is ambiguous. The discrepancy of the low-frequency E_g_ modes in the two experiments^14,16^ is more than 10 cm^−1^, and meanwhile both observed frequencies of the E_g_ modes are about 20–30 cm^−1^ higher than the theoretical result. Furthermore, the two high-frequency Raman active modes (B_1g_ and E_g_) have not been observed in all the experiments^14,16,37,38^ in spite of the high intensity of the B_1g_ mode in our calculations. The possible reason is due to the phonon damping caused by the large carrier concentration in Bi_2_O_2_Se, as indicated in Pereira’s work.^37^

**Table 4:** Comparison between calculated and experimental Raman frequencies of Bi_2_O_2_Se

	Raman frequency (cm^−1^)
This work	67.3 (E_g_), 162.9 (A_1g_), 354.3 (B_1g_), 433.3 (E_g_)
Experiment^14^	100, 159
Experiment^16^	84/90 (E^2^_g_), 159 (A_1g_)
Experiment^37^	159.2 (A_1g_)
Experiment^38^	160 (A_1g_)

The Raman spectrum of Bi_2_O_2_Te was also measured in Cheng’s work,^38^ which is listed in Table 5. The two observed Raman modes (A_1g_ and B_1g_) are well consistent with our calculations. However, the two E_g_ modes are not observed in their work. It is interesting to point out that the missing B_1g_ mode in Bi_2_O_2_Se was observed in Bi_2_O_2_Te, although in a relatively low intensity compared to that of the A_1g_ mode. Therefore, the Raman spectra of Bi_2_O_2_Se and Bi_2_O_2_Te need further investigations. For example, one could try to measure the Raman spectrum of Bi_2_O_2_Se with a lower carrier concentration by doping or at low temperatures in a proper Raman polarized configuration.

**Table 5:** Comparison between calculated and experimental Raman frequencies of Bi_2_O_2_Te

	Raman frequency (cm^−1^)
This work	69.1 (E_g_), 147.48 (A_1g_), 336.0 (B_1g_), 396.1 (E_g_)
Experiment^38^	147 (A_1g_), 340 (B_1g_)

### 3.3 *Pnnm* orthorhombic Bi_2_O_2_S

Although Bi_2_O_2_S has a very similar crystal structure to the one of Bi_2_O_2_Se shown in Fig. 1(a) and (b), they have a different symmetry. In fact, Bi_2_O_2_S has an orthorhombic crystal structure with a space group of *Pnnm* (point group *D*_2h_). There are ten atoms in the unit cell of Bi_2_O_2_S resulting in thirty phonon modes. Its irreducible representations at the *Γ* point are presented as follows:*Γ*_acoustic_ = B_1u_ + B_2u_ + B_3u_*Γ*_optic_ = 3A_u_ + 2B_1u_ + 5B_2u_ + 5B_3u_ + 4A_g_ + 4B_1g_ + 2B_2g_ + 2B_3g_The calculated zone-centered optical phonon frequencies of Bi_2_O_2_S and their symmetries are listed in Table 6. It is found that all of the modes are non-degenerate. According to the character table of the *D*_2h_ point group, the B_1u_, B_2u_, and B_3u_ modes are IR active, while the A_g_, B_1g_, B_2g_ and B_3g_ modes are Raman active. The A_u_ modes are neither IR nor Raman active. From our calculation, Bi_2_O_2_S should have twelve Raman and twelve IR active modes, as shown in Table 6.

**Table 6:** Mulliken symbols and frequencies of zone-centered optical phonon modes of Bi_2_O_2_S. Raman or IR activity of each mode is also indicated by “Raman” and “IR”. The A_u_ mode is neither Raman nor IR active. The unit of the phonon frequency is cm^−1^

Symmetry	Bi_2_O_2_S	Activity	Symmetry	Bi_2_O_2_S	Activity
B_2g_	9.5	Raman	B_3u_	218.8	IR
A_g_	13.9	Raman	A_g_	285.2	Raman
A_u_	53.7		B_3u_	286.3	IR
B_2u_	54.9	IR	B_2g_	287.7	Raman
B_3u_	60.7	IR	B_1u_	288.5	IR
B_1u_	64.9	IR	B_3u_	364.4	IR
B_3g_	65.2	Raman	A_g_	367.8	Raman
B_1g_	67.9	Raman	B_2u_	404.2	IR
B_2u_	75.7	IR	A_u_	448.9	
B_1g_	83.0	Raman	B_1g_	450.6	Raman
A_u_	113.1		B_3g_	452.4	Raman
B_2u_	113.4	IR	B_2u_	457.5	IR
B_3u_	144.7	IR	B_1g_	519.1	Raman
A_g_	170.6	Raman			

The vibrational eigenvectors of all the zone-centered optical modes and the polarized configurations of the Raman active modes are shown in Fig. S1 and Tables S1 and S2 in the ESI.†

**Fig. 4:**
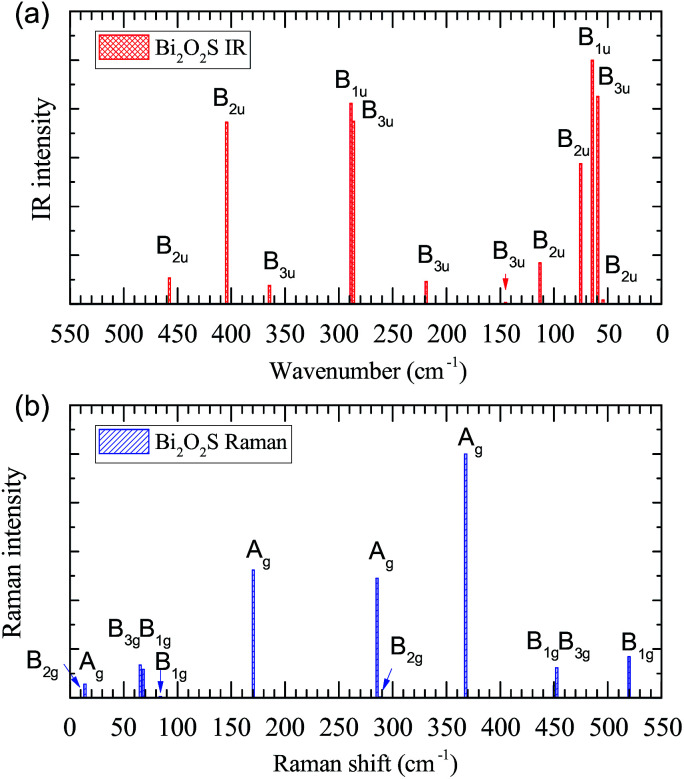
Calculated IR and Raman intensities (arbitrary unit) of orthorhombic Bi_2_O_2_S.

IR and Raman intensities of Bi_2_O_2_S are also calculated directly by first principles calculations, which are shown in Fig. 4. It is found that the IR modes near 60, 290 and 400 cm^−1^ have the highest intensities. In the Raman spectrum, the three A_g_ modes near 170, 285, and 370 cm^−1^ have the highest intensities.

It is noted that Bi_2_O_2_S has been synthesized in experiments,^24–26^ however no Raman spectrum was measured yet. Recently, Cheng *et al.* have also calculated the Raman spectrum of Bi_2_O_2_S by the density functional perturbation theory in the local density approximation and norm-conserving pseudopotentials implemented in Quantum Espresso (QE) software.^38^ We listed their data in Table 7 as well as ours for comparison. From the frequency perspective, we can see that the two calculations are in general consistent with each other. For example, in both works, there are five Raman modes below 100 cm^−1^, one mode between 100–200 cm^−1^, two modes between 200–300 cm^−1^, and *etc.* Although the largest difference in a B_1g_ mode reaches 43 cm^−1^ (about 10%), we still think it is acceptable since the two works use totally different methods in their calculations.

**Table 7:** Comparison between the theoretical Raman frequencies of Bi_2_O_2_S. For each row, the Raman modes are arranged according to their frequencies. The unit of the phonon frequency is cm^−1^

This work	B_2g_	A_g_	B_3g_	B_1g_	B_1g_	A_g_	A_g_	B_2g_	A_g_	B_1g_	B_3g_	B_1g_
	9.5	13.9	65.2	67.9	83.0	170.6	285.2	287.7	367.8	450.6	452.4	519.1
Cheng^38^	B_2g_	A_g_	B_2g_	A_g_	A_g_	A_g_	B_1g_	B_3g_	B_1g_	B_1g_	B_3g_	B_1g_
	20.52	29.23	64.34	68.23	82.86	154.20	263.85	273.27	386.85	407.76	417.30	520.28

However, we noted that the Mulliken symbols in the two works are quite different. In particular, the four A_g_ modes in Cheng’s work are all below 200 cm^−1^, while we have two A_g_ modes below 200 cm^−1^ and two other ones above 200 cm^−1^. The highest A_g_ mode in our work is more than 210 cm^−1^ higher than theirs. This cannot be explained by the inaccuracy of the phonon frequency induced by the different parameters. It is possibly due to the different classification of the Mulliken symbols. In the *D*_2h_ point group, the assignment of B_1g_, B_2g_, and B_3g_ depends on the three mutually perpendicular 2-fold axes along the *z*, *y*, and *x* directions.^56^ We have tested that QE software will give different Mulliken symbols (B_1g_, B_2g_, and B_3g_) depending on the orientations of the orthorhombic unit cell of Bi_2_O_2_S. However, the assignment of the A_g_ mode should be unambiguous, which is independent of the directions of the unit cell. Therefore, we think the discrepancy of the A_g_ Raman modes in our work and Cheng’s work needs further investigations.

### 3.4 *P*4/*nmm* tetragonal Bi_2_OS_2_, Bi_2_OSe_2_, and Bi_2_OTe_2_

In experiment, Bi_2_OS_2_ has a space group of *P*4/*mmm* (point group *D*_4h_).^28,55^ However, to the best of our knowledge, Bi_2_OSe_2_ and Bi_2_OTe_2_ have not been synthesized in experiment. First principles calculations indicate that they share the same crystal structure as Bi_2_OS_2_.^30^ There are ten atoms in the unit cell of Bi_2_OX_2_ (X = S, Se, and Te) as shown in Fig. 1(c), resulting in thirty phonon modes. The irreducible representations of Bi_2_OX_2_ at the *Γ* point are:*Γ*_acoustic_ = E_u_ + A_2u_*Γ*_optic_ = 4E_u_ + 4A_2u_ + 5E_g_ + 4A_1g_ + B_1g_The zone-centered optical phonon frequencies and their symmetries of Bi_2_OX_2_ are listed in Table 8. The vibrational eigenvectors of Bi_2_OS_2_ are shown in Fig. S2 in the ESI.† The polarized configurations of the Raman spectra of Bi_2_OX_2_ should be the same as those of Bi_2_O_2_Se (Table 3) since they all belong to the *D*_4h_ point group.

**Table 8:** Mulliken symbols and frequencies of zone-centered optical phonon modes of Bi_2_OX_2_ (X = S, Se and Te). Raman or IR activity of each mode is also indicated by “Raman” and “IR”. The unit of the phonon frequency is cm^−1^

Symmetry	Bi_2_OS_2_	Bi_2_OSe_2_	Bi_2_OTe_2_	Activity
E_u_	26.0	18.8	6.5	IR
E_g_	30.7	25.9	20.0	Raman
E_g_	63.2	55.8	41.1	Raman
A_2u_	63.9	54.6	50.6	IR
A_1g_	73.2	64.1	53.5	Raman
E_u_	97.7	73.2	49.2	IR
E_g_	111.4	80.5	54.8	Raman
E_u_	126.6	89.4	83.5	IR
A_2u_	129.4	97.2	84.5	IR
A_1g_	132.2	88.6	75.1	Raman
E_g_	138.4	100.2	101.1	Raman
A_1g_	149.7	138.6	123.9	Raman
E_u_	262.1	228.1	183.7	IR
A_2u_	286.0	182.3	140.9	IR
A_1g_	346.5	217.9	163.2	Raman
B_1g_	363.5	342.2	311.2	Raman
E_g_	415.0	376.0	321.9	Raman
A_2u_	466.0	420.0	372.1	IR

According to the character table for the *D*_4h_ point group, the E_u_ and A_2u_ modes are IR active, while the E_g_, A_1g_, and B_1g_ modes are Raman active. Therefore, there are ten Raman active (five double degenerated E_g_ modes, five non-degenerated A_1g_ and B_1g_ modes) and eight IR active modes (four double degenerated E_u_ modes and four non-degenerated A_2u_ ones) in Bi_2_OX_2_.

**Fig. 5:**
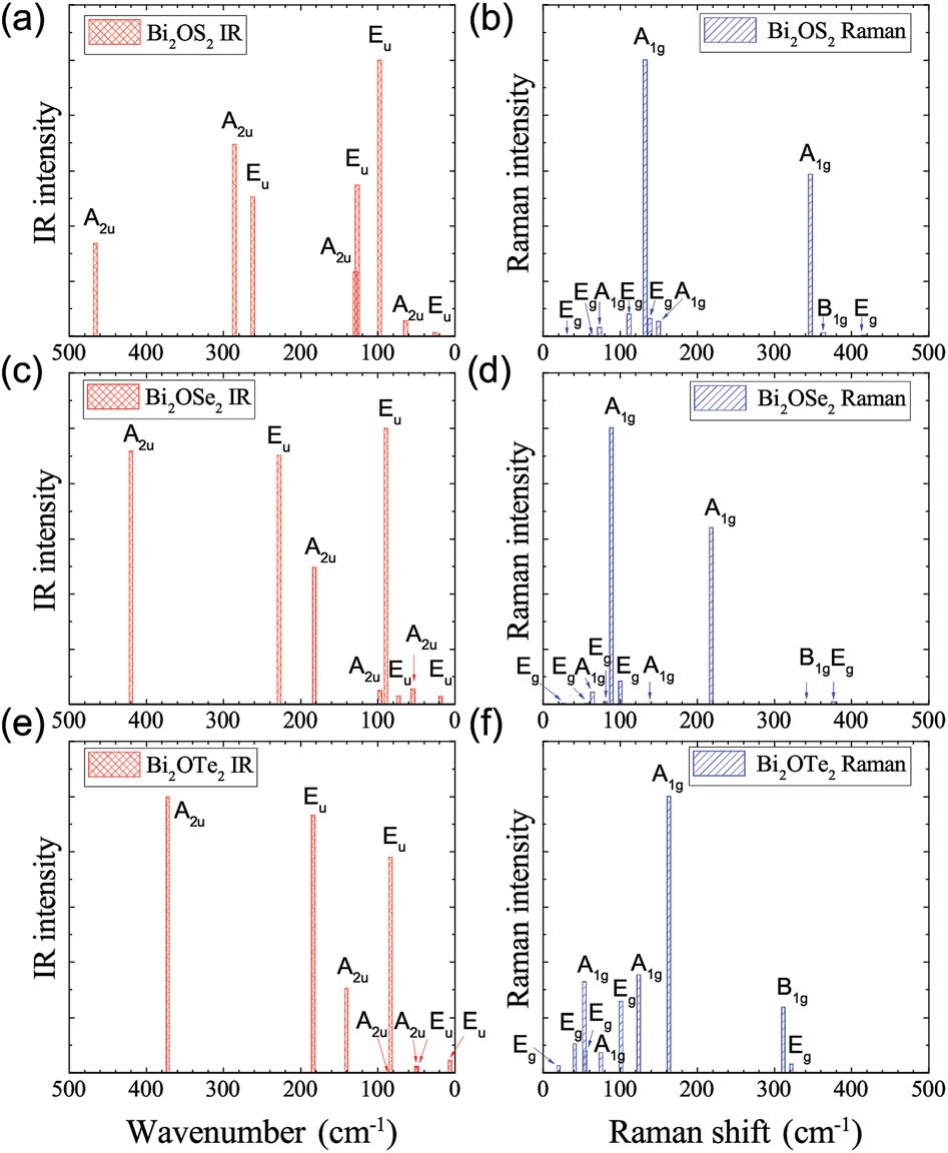
Calculated IR and Raman intensities (arbitrary unit) of tetragonal Bi_2_OX_2_ (X = S, Se, and Te).

The IR and Raman intensities of Bi_2_OX_2_ are also calculated directly by first principles calculations, which are shown in Fig. 5. It is found that in the IR spectrum of Bi_2_OS_2_, there are six modes (E_u_ modes around 98, 127, 262 cm^−1^ and A_2u_ modes around 129, 286, 466 cm^−1^) which have relatively high intensities. For the Bi_2_OSe_2_ and Bi_2_OTe_2_, only four modes have high intensities. For the Raman spectra of Bi_2_OS_2_ and Bi_2_OSe_2_, there are two promising A_1g_ peaks around 132 and 346 cm^−1^ for Bi_2_OS_2_, and 89 and 218 cm^−1^ for Bi_2_OSe_2_. For Bi_2_OTe_2_, the A_1g_ Raman mode around 163 cm^−1^ has the highest intensity.

Since the tetragonal Bi_2_OSe_2_ and Bi_2_OTe_2_ have not been synthesized in experiments, we also calculated their phonon dispersion and densities of state, which are not shown here. No imaginary frequencies are found in both materials. Therefore we think the tetragonal phases of Bi_2_OSe_2_ and Bi_2_OTe_2_ are stable and they could possibly be synthesized in future experiments.

## 4 Conclusions

We have systematically calculated the Raman and infrared spectra of six Bi–O–X materials: Bi_2_O_2_X and Bi_2_OX_2_ (X = S, Se, and Te). For each material, we present their optical phonon frequencies, Raman and infrared activities and intensities, Raman polarization configurations, and vibrational eigenvectors. In particular, the Raman spectra of Bi_2_O_2_Se and Bi_2_O_2_Te are compared with the existing experimental results. In Bi_2_O_2_Se, only one A_1g_ Raman mode is confirmed in experiments, while the other three are ambiguous or not observed yet. In Bi_2_O_2_Te, both A_1g_ and B_1g_ modes are well consistent with the experiments, while two E_g_ modes are not observed yet. Due to the various and important physical properties in these materials, our work could be helpful in identifying the crystal structure in future experiments.

## Conflicts of interest

There are no conflicts to declare.

## Supplementary Material

RA-009-C9RA02584G-s001
