# Supplementary material for: Infrared and Raman spectra of Bi2O2X and Bi2OX2 (X = S, Se, and Te) studied from first principles calculations
Source: RSC Adv. 2019 Jun 10;9(31):18042–9. doi: 10.1039/c9ra02584g (PMC9064694; doi:10.1039/c9ra02584g)
Supplement: RA-009-C9RA02584G-s001 [file RA-009-C9RA02584G-s001.pdf]

Electronic Supplementary Material (ESI) for RSC Advances.
This journal is © The Royal Society of Chemistry 2019

Supplementary Information for "Infrared and Raman spectra of Bi_2O_2X and Bi_2OX_2 (X=S, Se, and Te) studied from first principles calculations"

Yaodi Xu[1], Cong Wang[1], Yang-Yang Lv[1], Y. B. Chen[2], Shu-Hua Yao[1,3,4], and Jian Zhou[1,3,4]*

[1] *National Laboratory of Solid State Microstructures and Department of Materials Science and Engineering, Nanjing University, Nanjing 210093, China*

[2] *National Laboratory of Solid State Microstructures and Department of Physics, Nanjing University, Nanjing 210093, China*

[3] *Collaborative Innovation Center of Advanced Microstructures, Nanjing University, Nanjing , 210093, China.*

[4] *Jiangsu Key Laboratory of Artificial Functional Materials, Nanjing University, Nanjing 210093 China*

(Dated: January 21, 2019)

PACS numbers:

*Corresponding author: zhoujian@nju.edu.cn

TABLE S1: The right angle scattering polarized configurations for the Raman active modes of orthorhombic Bi_2O_2S.

Configuration	A_g	B_{1g}	B_{2g}	B_{3g}
X(YY)Z	√			
X(ZZ)Y	√			
Y(XX)Z	√			
Y(ZZ)X	√			
Z(XX)Y	√			
Z(YY)X	√			
Y(XY)X		√		
Y(XY)Z		√		
Z(XY)X		√		
Z(XZ)X			√	
Z(XZ)Y			√	
Y(XZ)X			√	
X(YZ)Y				√
Z(YZ)X				√
Z(YZ)Y				√

TABLE S2: The back scattering polarized configurations for the Raman active modes of orthorhombic Bi_2O_2S.

Configuration	A_g	B_{1g}	B_{2g}	B_{3g}
-X(YY)X	√			
-X(ZZ)X	√			
-Y(XX)Y	√			
-Y(ZZ)Y	√			
-Z(XX)Z	√			
-Z(YY)Z	√			
-Z(XY)Z		√		
-Y(XZ)Y			√	
-X(YZ)X				√

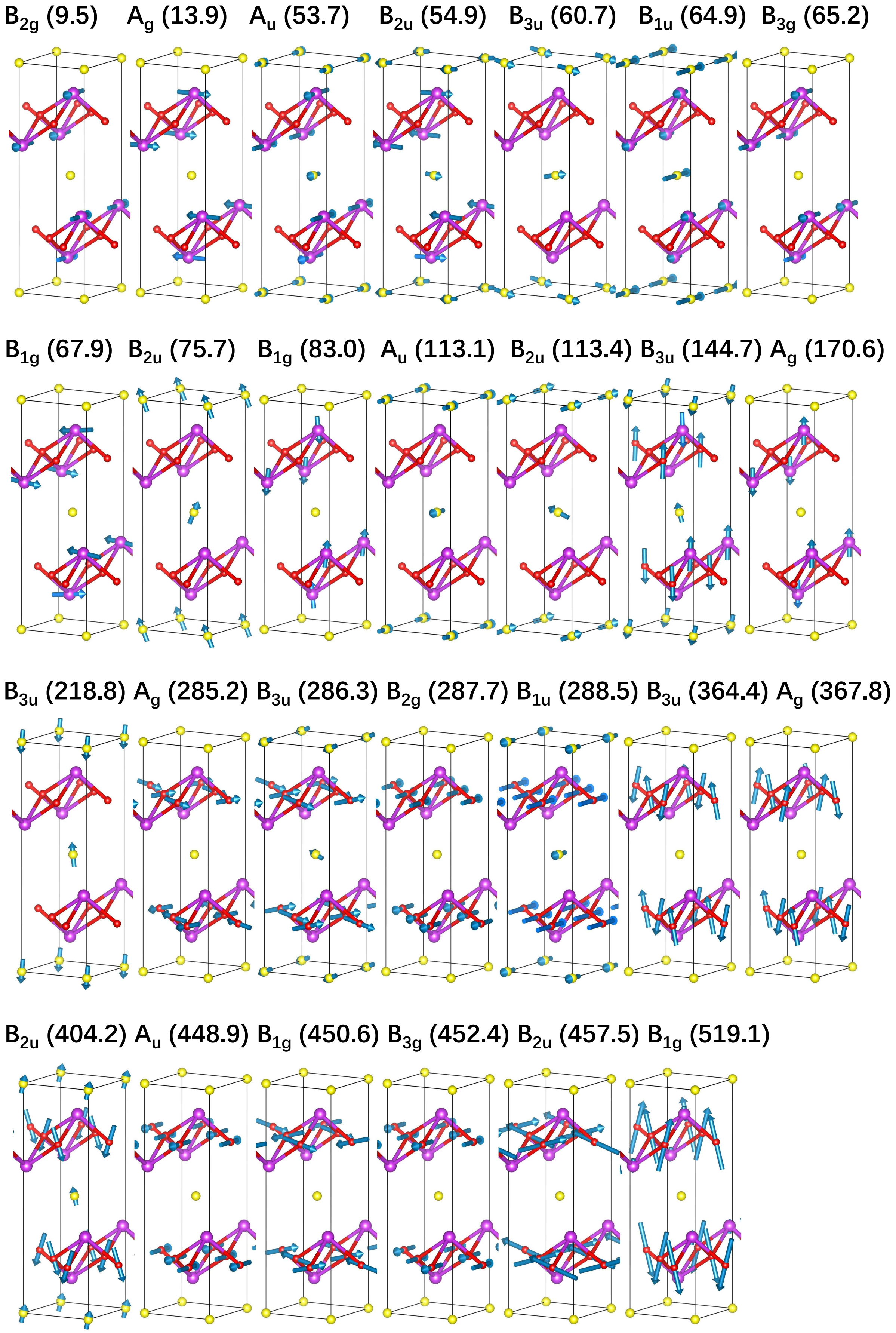

FIG. S1: Vibrational eigenvectors and Mulliken symbols of the zone-centered optical phonon modes shown in an orthorhombic Bi_2O_2S (Pnnm). The purple, red, and yellow balls represent Bi, O, and S atoms respectively.

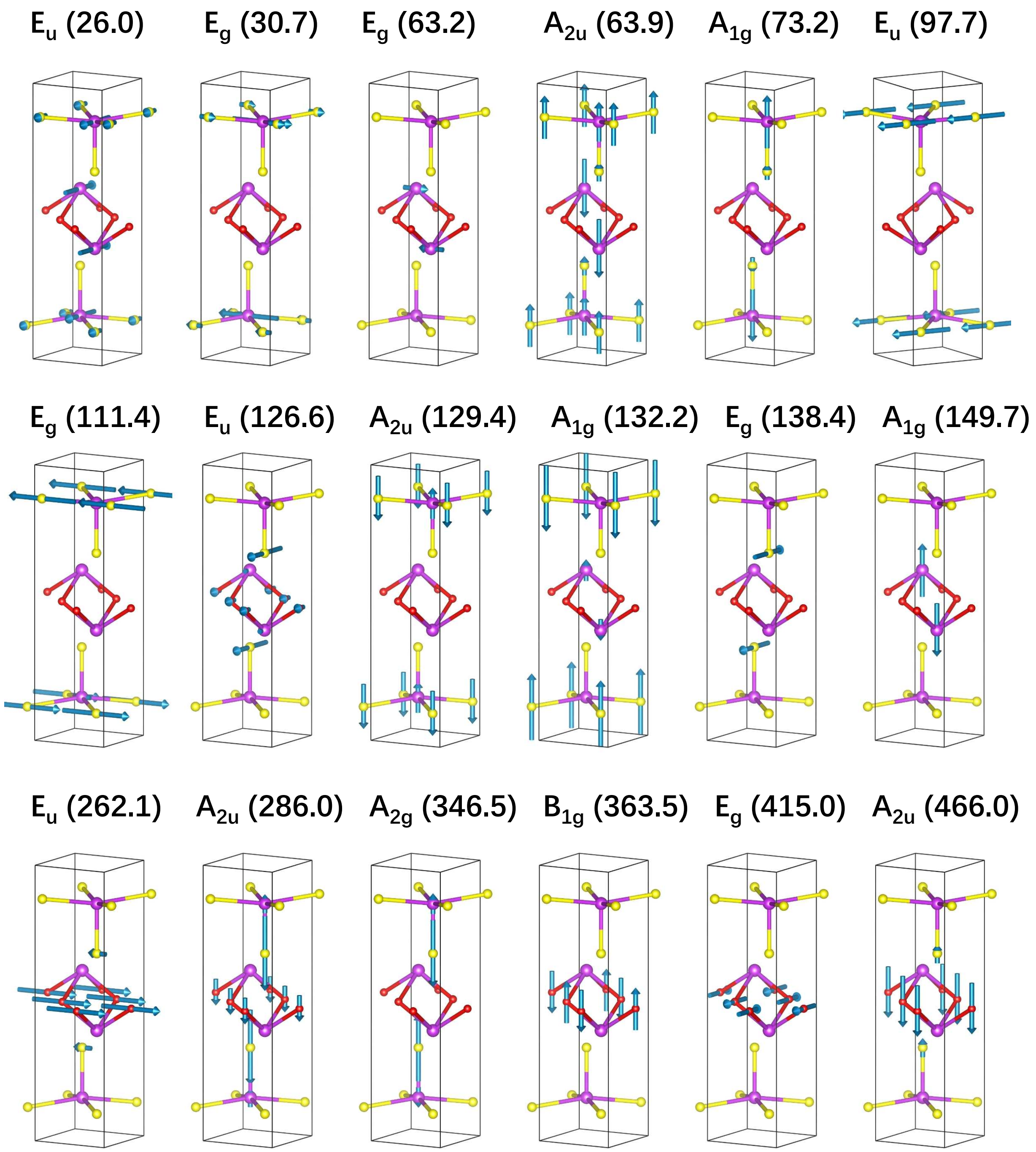

FIG. S2: Vibrational eigenvectors and Mulliken symbols of the zone-centered optical phonon modes shown in a tetragonal Bi_2OS_2 (P4/nmm). The purple, red, and yellow balls represent Bi, O, and S atoms respectively.
